# Autopsies at Central Park Menagerie

**Published:** 1887-01

**Authors:** 


### CASE DEPARTMENT.

### AUTOPSIES AT CENTRAL PARK MENAGERIE.

SPOTTED HYENA—*Hyæna Crocuta*, *Hab.* South Africa, died November 23d, 1886. It had fed nearly as well as usual, on the day of its death, but had moved about the cage as if cramped up in its abdomen during the last few days.

On opening the thoracic and abdominal cavities, an enormous sac was observed, crowding up the diaphragm and almost completely filling the abdomen. It extended to the *symphysis pubis*, contained fully two gallons, and measured over eighteen inches in the sagittal direction, and extended from the right to the left false ribs in the transverse diameter. This enormous sac was the stomach. Closer examination revealed the existence of a *duodenal stricture.* The seat of the stricture showed the usual characters of the intestinal tunic, except at one point, where a mass of almost cartilaginous hardness was found, which consisted of fibrillar connective tissue. The pyloric orifice had been greatly distended, obliterating the valve. A conical passage led to the site of the stricture, gradually narrowing down to the size of a crow quill before reaching the latter. The stricture admitted a bristle with difficulty, and did not permit fluid to pass through without considerable pressure. It was slightly twisted, the twist being evidently due to the distorting influence of the expanding stomach.

The intestinal canal was remarkably atrophied, and crowded into the pelvic cavity; it could have been held in the hollow of one hand. Its diameter was in few places more than one centimeter. The texture was normal, though slightly atrophic, and the color intensely green, owing to the fact that the contents were exclusively bile, which had an uninterrupted flow into the intestinal canal immediately below the stricture.

The stomach had an enormously hypertrophied mucosa. The ridges and folds were greatly magnified, forming a sort of reticulum. In the meshes of this reticulum the muscular coat was in part atrophic. There were some slight hemorrhages into the mucus covering the folds, but neither ulceration nor signs of true inflammation could be discovered. The contents of the stomach were meat evidently eaten before death, bones, softened to gristle-like masses, some of them several inches in diameter, and *stercoraceous* material of a dark tea-leaf color. As a regurgitation of bile was impossible, this is an interesting observation.

The heart was in a flaccid condition, though like the lungs, kidneys, central nervous system, etc., it was texturally remarkably healthy, as indeed the animal was, aside from the gastric dilatation and stricture, one of the healthiest we have seen.

Anatomical Diagnosis: Stricture of the duodenum, gradual occlusion at the seat of stricture, secondary dilatation of stomach, torsion ensuing in consequence and complete closure. The probable cause of death was reflex paralysis of the heart. E. C. S.

HORSE DISEASE AT COONONG.—Mr. Edward Stanley, F.R.C.V.S., has forwarded to the Chief Inspector of Stock a report on the outbreak of disease among horses feeding on ensilage. The symptoms were in all cases alike, varying only in degree. They were listlessness, prostration of strength, sore throat (without swelling or pain upon manipulation). Irritation was shown by liquids being swallowed with great difficulty, although thirst was marked. Food and water were both returned through the nostrils, this often being accompanied by fits of coughing. The bowels were constipated, with straining; the dung was dark-colored and most offensive. The urine was passed freely, and in large quantities; it was of the usual color and clear. It presented nothing diagnostic on examination. The pulse was small, hard, feeble, and indistinct. The heart was irritable and labored, ranging from 40 to 60 beats per minute. Respiration was slightly accelerated; but with feeble chest and abdominal movements, visible mucous membranes were of a dirty, brownish-yellowish color. The tongue was pasty and dry, emitting a horrid, nauseating odor, almost unbearable. The temperature ranged from 95 to 100 degrees. The extremities were cold. The *post-mortem* appearances were as follows: Congestion of the nasal passages, larynx, and trachèa. The tongues were dry and thickly furred. The pharynx was inflamed, but free from ulceration or diptheritic deposit. The stomachs and large intestines were of a brownish-black color, the mucous membrane being deeply stained. Contents of the rectum, hard and dry. The peritoneum was stained in large patches of a ruddy color, and the mesenteric veins were filled with black, coagulated blood. The bladders were ecchymosed in a most remarkable manner. Heart was flabby, dark in color. Extravasations of blood occurred along the blood-vessels, and also on the fleshy pillars inside the ventricles. Both right and left cavities contained firm clots of blood, separated by gravitation. Liver was dark and slightly ecchymosed. Spleen healthy in appearance. Lungs, in one case, were full of congested blood, and the treachæ and bronchial tubes were greenish black in color. The unfortunate fatality led Mr. Stanley to the conclusion that ensilage exposed to the air for a very few days under favorable climatic conditions, such as moisture and temperature heat, forms an excellent seed-bed and nourishing medium for fungoid growths, such as moulds and low forms of vegetable life. Their germs being always present in the air, are increased to myriads under favorable conditions, and such undoubtedly existed in this outbreak. Rain having recently fallen, following a long period of heat and drought, and the horses being weak, predisposed them to the ill effects of the fungus, which not only entered their systems through the stomach, but also during respiration. Being exposed to the same poisonous agents day after day, chronic poisoning and fatal consequences followed. W. A. C.

Cerebro-Spinal Meningitis of Cattle.—At the last congress of German Naturalists and Physicians, held at Berlin, Dr. Schmidt, of Aix-la-Chapelle, described the hitherto imperfectly recognized disease of cattle known as cerebro-spinal meningitis. There is no change of temperature, pulse, or respiration but during the stage of excitation, during which the animals are restless and throw the head from side to side, but are unable to flex or extend it. This is followed by a stage of depression; there is a tonic spasm of the muscles of the neck; there may be trismus, and the animal, although not stupefied, is very lethargic. The skin becomes cool, the general temperature sinks to 38 degrees C, and regularly, from the second day on, there are spasms of the longissimus dorsi, clonic, recurring about twenty times a minute. Spasms in other muscles, such as the serratus and those of the eye-lids, sometimes occur, and gradually increase in intensity. Occasionally the fore-leg is paralyzed. Only one patient recovered, and this one had the muscular spasms four weeks. All other cases terminated fatally, in from six to eight days. On the autopsy, in early cases, hyperæmia of the brain membranes was found; in those killed on the second to fourth day, a clear fluid was found in the meshes of the pia; and in later ones, a gelatinous material, particularly in the sulci of the cerebrum. The ventricles, as well as the tissue proper of the brain and cord, appeared normal. E. C. S.

